# Tailoring the Surface of Polylactide/Poly(itaconate derivative) Fibers: A Path to Antibacterial Enhancement

**DOI:** 10.1021/acsomega.6c04582

**Published:** 2026-07-28

**Authors:** Jakub Zágora, Zuzana Rybková, Kateřina Škrlová, Kateřina Malachová, Alexandra Muňoz-Bonilla, Marta Fernández-Garcia, Daniela Plachá

**Affiliations:** † Nanotechnology Centre, CEET, VSB-Technical University of Ostrava, 17. listopadu 2172/15, 708 00 Ostrava-Poruba, Czech Republic; ‡ Faculty of Materials Science and Technology, VSB-Technical University of Ostrava, 17. listopadu 2172/15, 708 00 Ostrava-Poruba, Czech Republic; § Department of Biology and Ecology, Faculty of Science, 48300 University of Ostrava, Chittussiho 10, 71000 Ostrava, Czech Republic; ∥ Department of Chemical Biology, Faculty of Science, Palacký University Olomouc, 17. listopadu 1192/12, 779 00 Olomouc, Czech Republic; ⊥ Institute of Science and Technology of Polymers, CSIC, Juan de la Cierva 3, 28000 Madrid, Spain; # CATRIN-RCPTM, Palacký University Olomouc, Šlechtitelů 241/27, 779 00 Olomouc, Czech Republic; ∇ Department of Epidemiology and Public Health, Faculty of Medicine, University of Ostrava, Syllabova 19, 703 00 Ostrava-Vítkovice, Czech Republic

## Abstract

Polylactide (PLA) is a biodegradable polymer with satisfactory mechanical properties but lacks antimicrobial activity. In this work, PLA fibers were functionalized with a cationic derivative of poly­(itaconic acid) (PTTIBuI) and treated using supercritical carbon dioxide (scCO_2_). Electrospun PLA and PLA + PTTIBuI fibers were subjected to scCO_2_ treatment at 200 bar and 60 °C for 3 and 6 h and subsequently characterized. Surface analysis revealed wrinkling, crystallization, increased crystallinity, and reduced hydrophobicity after treatment. Antimicrobial testing showed that PLA + PTTIBuI fibers significantly inhibited biofilm formation, with stronger effects against *Acinetobacter baumannii* (50–60%) than against methicillin-resistant *Staphylococcus aureus* (43–55%). Long-term scCO_2_ exposure increased antibacterial activity, likely due to increased surface accessibility of cationic groups. These results demonstrate that the combination of poly­(itaconic acid)-based antimicrobial agents with supercritical fluid treatment is an effective strategy for tailoring PLA fibers with improved antimicrobial activity. The modified materials show strong potential for biomedical applications such as wound dressings and infection prevention agents.

## Graphical abstract

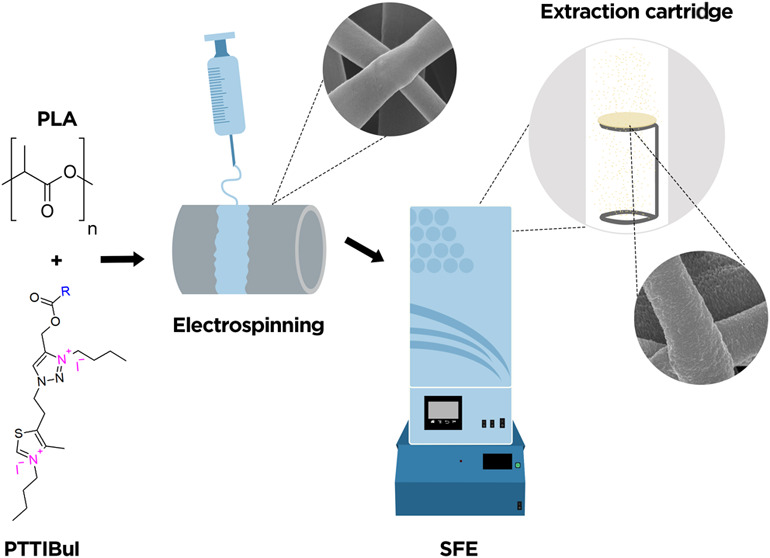

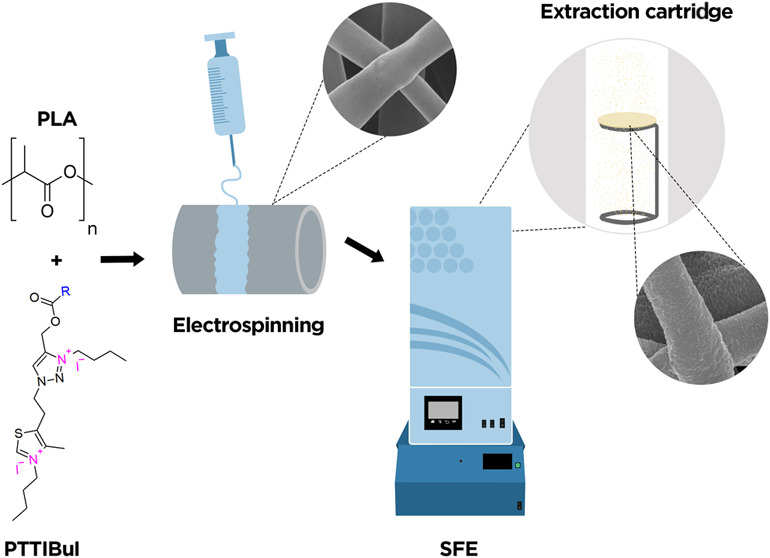

## Introduction

The use of biomaterials in wound treatment has gained attention due to their ability to facilitate healing and reduce infection risks.^1^ Antimicrobial materials, such as silver nanoparticles,^2^ chitosan,^3,4^ and antimicrobial peptides,^5^ play a crucial role in preventing and treating infections by integrating antimicrobial agents into biomaterials. These materials enhance the efficacy of wound dressings and promote faster healing.^6–8^ Polylactide (PLA) is valued in biomedicine for its biocompatibility, degradation kinetics, and mechanical strength but lacks inherent antimicrobial properties, limiting its wound treatment applications. The hydrophobic nature of PLA also hinders cellular adhesion and proliferation, which challenges tissue regeneration.^2,9–11^ Various modification strategies, including incorporating antimicrobial fillers and blending with antimicrobial polymers, aim to overcome these limitations.^2–14^ Methacrylate-derived polymers are versatile and biocompatible, suitable for drug delivery and tissue engineering but may cause irritation and cytotoxicity.^15–17^ Natural-based polymers like itaconic acid offer safer alternatives with biocompatibility, biodegradability, and immunomodulatory properties, suitable for wound treatment.^18,19^

Supercritical fluids, especially CO_2_ (scCO_2_), are effective in modifying fibers due to their unique properties as solvents.^20,21^ Compared to other physical methods, the supercritical process does not degrade the fibers, requires less energy due to the lower processing temperature, and uses CO_2_, which can be regenerated and recycled.^22–24^ Unlike conventional PLA surface-modification methods such as (i) high-energy irradiation, which induces chain scission and molecular weight loss,^25,26^ (ii) plasma treatments, whose effect is shallow and prone to hydrophobic recovery; and (iii) wet-chemical routes, which use corrosive reagents and erode the surface, supercritical CO_2_ is nonionizing, nontoxic, operates at a moderate temperature, and modifies polymers surface without degrading the polymer chain.^27,28^

This work aims to develop microsized fibers from PLA and modify them with modified itaconic acid–based antimicrobial polymers, using supercritical fluid technology to enhance their antimicrobial properties for wound treatment without additional additives (e.g., peptides, essential oils).^5,29,30^ The fibers were created from a combination of itaconic acid–based polymers and PLA,^31^ then processed with supercritical fluid technology. The modified fibers showed changes in their morphology and antimicrobial activity, making them suitable for air filtration, medical bandages, and masks.

## Materials and Methods

### Materials

This part describes the synthesis of poly­(5,5′-(((((2-methylenesuccinyl) bis­(oxy)) bis­(methylene)) bis­(3-butyl-1*H*-1,2,3-triazol-3-ium-4,1-diyl)) bis­(ethane-2,1-diyl)) bis­(3-butyl-4-methylthiazol-3-ium)) iodide (PTTIBuI), an antimicrobial compound synthesized from natural itaconic acid.^32^ This compound demonstrates biodegradability without producing any harmful metabolites during its degradation.^31–33^

The following chemicals were used to synthesize the antimicrobial polymer: 4-(dimethylamino)­pyridine (DMAP, ≥99%), propargyl alcohol (≥99%), itaconic acid (IA, ≥99%), hydroquinone (≥99%), *N*,*N*,*N*′,*N*″,*N*‴-pentamethyldiethylenetriamine (PMDETA, ≥99%), *N*,*N*′-dicyclohexylcarbodiimide (DCC, ≥99%), copper chloride (CuCl, ≥99.995%), 1-iodobutane (BuI, ≥99%), ammonium persulfate (APS, ≥98%), methylsulfonyl chloride (MsCl) (>99.7%), 5-(2-hydroxyethyl)-4-methylthiazole (>98%), sodium azide (NaN_3_), magnesium sulfate anhydrous (MgSO_4_, ≥99,5%), sodium bicarbonate (NaHCO_3_, ≥99.7%), neutral aluminum oxide were purchased from Sigma-Aldrich. Anhydrous *N*,*N*-dimethylformamide (DMF, ≥99.8%), anhydrous tetrahydrofuran (THF, ≥99.9%), dichloromethane (CH_2_Cl_2_, ≥99%), methanol (MeOH, ≥99%), hexane (≥99%), chloroform (≥99%), triethylamine (TEA), ethanol (EtOH, ≥99%) were purchased from Scharlau. 2,2′-azobis­(isobutyronitrile) (AIBN, ≥98%) for initiation was taken at Acros. Before use, it was recrystallized twice from methanol. Toluene (≥99%) was purchased from Merck, sulfuric acid (H_2_SO_4_) from Panreac and ethyl acetate (EtOAc, ≥99%) from Cor. Quimica S.L. Carbon dioxide (CO_2_) (4.8) from SIAD, Czech Republic was used for supercritical processes. Polylactide (PLA) Ingeo 4032D was received from RESINEX Czech Republic s.r.o. with the characteristic properties: Mw 182,000 g mol^–1^, density 1.24 g/cm^3^, glass transition temperature (*T*_g_) 59 °C and melting point 160 °C.^2,11^

### Preparation of PTTIBuI

The poly­(5,5′-(((((2-methylenesuccinyl) bis­(oxy)) bis­(methylene)) bis­(3-butyl-1H-1,2,3-triazol-3-ium-4,1-diyl)) bis­(ethane-2,1-diyl)) bis­(3-butyl-4-methylthiazol-3-ium)) iodide (PTTIBuI) was synthesized following literature conditions.^32,34^ Briefly, the six-step synthesis comprised the preparation of 5-(2-azidoethyl)-4-methylthiazole from 5-(2-hydroxyethyl)-4-methylthiazole in two steps, followed by a three-step preparation of poly­(bis­((1-(2-(4-methylthiazol-5-yl)­ethyl)-1*H*-1,2,3-triazol-4-yl)­methyl) itaconate) and a final quaternization.

In the first step, 62.7 mL (534 mmol) of 5-(2-hydroxyethyl)-4-methylthiazole was dissolved in 250 mL of CH_2_Cl_2_ (anhydrous) at 0 °C. 78 mL (593 mmol) of freshly distilled TEA was added to the solution, followed by 43 mL (593 mmol) of MsCl, added dropwise over the course of 1 h at the same temperature. The reaction mixture was then stirred at room temperature for 4 h, after which 250 mL of a saturated aqueous NaHCO_3_ solution was added. The mixture was then transferred to a separatory funnel, where the organic layer was separated and the aqueous phase was further extracted with 2 × 100 mL of CH_2_Cl_2_. All three organic layers were combined, dried over anhydrous MgSO_4_, filtered, and concentrated on a vacuum rotary evaporator, yielding 2-(4-methylthiazol-5-yl)­ethylmethanesulfonate denoted as M1.^11,34^

In the second step, 100.0 g (452 mmol) of 2-(4-methylthiazol-5-yl)­ethylmethanesulfonate was dissolved in 1000 mL of DMF (anhydrous). 73.5 g (1130 mmol) of NaN_3_ was added portionwise to this solution. The mixture was stirred for 4 h at 70 °C and then cooled to 25 °C. The solvent was evaporated on a vacuum rotary evaporator, the resulting product was dissolved in 500 mL of EtOAc/H2O (1:1). The organic layer was then separated and washed 3 times with 200 mL of H_2_O. The organic layers were combined and dried over anhydrous MgSO_4_, filtered and concentrated under reduced pressure to give a dark orange oil, which was purified by column chromatography (hexane/EtOAc, 3:1 to 1:1) to give product M2 as a clear yellow oil.^11,32,34^

The monomer was then prepared by dissolving 15 g of itaconic acid, 32.3 g of propargyl alcohol and 1.26 g of hydroquinone in 50 mL of THF at 60 °C in a flask equipped with a Dean–Stark trap. Then 250 mL of toluene and 300 μL of H_2_SO_4_ were added and the mixture was heated under reflux for 24 h. During this time, a total of 4 mL of water was removed. Then the solvents were partially evaporated in vacuo and the mixture was washed with saturated NaHCO_3_. The organic extract was dried over anhydrous MgSO_4_, filtered and purified through a neutral alumina column using hexane/EtOAc (1:1) to give di­(prop-2-yn-1-yl)­itaconate (PrI).^32^

PrI was then radically polymerized to obtain poly­(di­(prop-2-yn-1-yl) itaconate) (PPrI). A 2 M solution of PrI in anhydrous DMF was prepared at 70 °C, AIBN (0.16 g) was added as the initiator. The mixture was deoxygenated using argon and stirred at 70 °C for 24 h. Then, the polymer was isolated, precipitated in ethanol, collected, and dried.

In the next step, PPrI (1.60 g), 5-(2-azidoethyl)-4-methylthiazole (M2, 1.51 g), PMDETA (312 μL), and 0.05 g of CuCl were dissolved in 40 mL of CHCl_3_. The reaction mixture was stirred at room temperature for 24 h and then passed through a column packed with neutral alumina to remove residual copper. The product (PTTI) was purified by column chromatography using a gradient of hexane (1:1 to 1:3) followed by ethanol and dried to give a white powder.^32^

Finally, PTTI was modified by N-alkylation with 1-iodobutane (BuI) to obtain the cationic polymer. 1.5 g of PTTI was dissolved in 25 mL of anhydrous DMF. Then 2.7 mL of BuI was added, and the mixture was degassed using argon, sealed, and stirred at 70 °C for 1 week. The final product was purified by precipitation in hexane, dialyzed, and freeze-dried to give PTTIBuI as a purple-brown powder. The entire reaction scheme is shown in Figure 1.^32^

**Figure 1:**
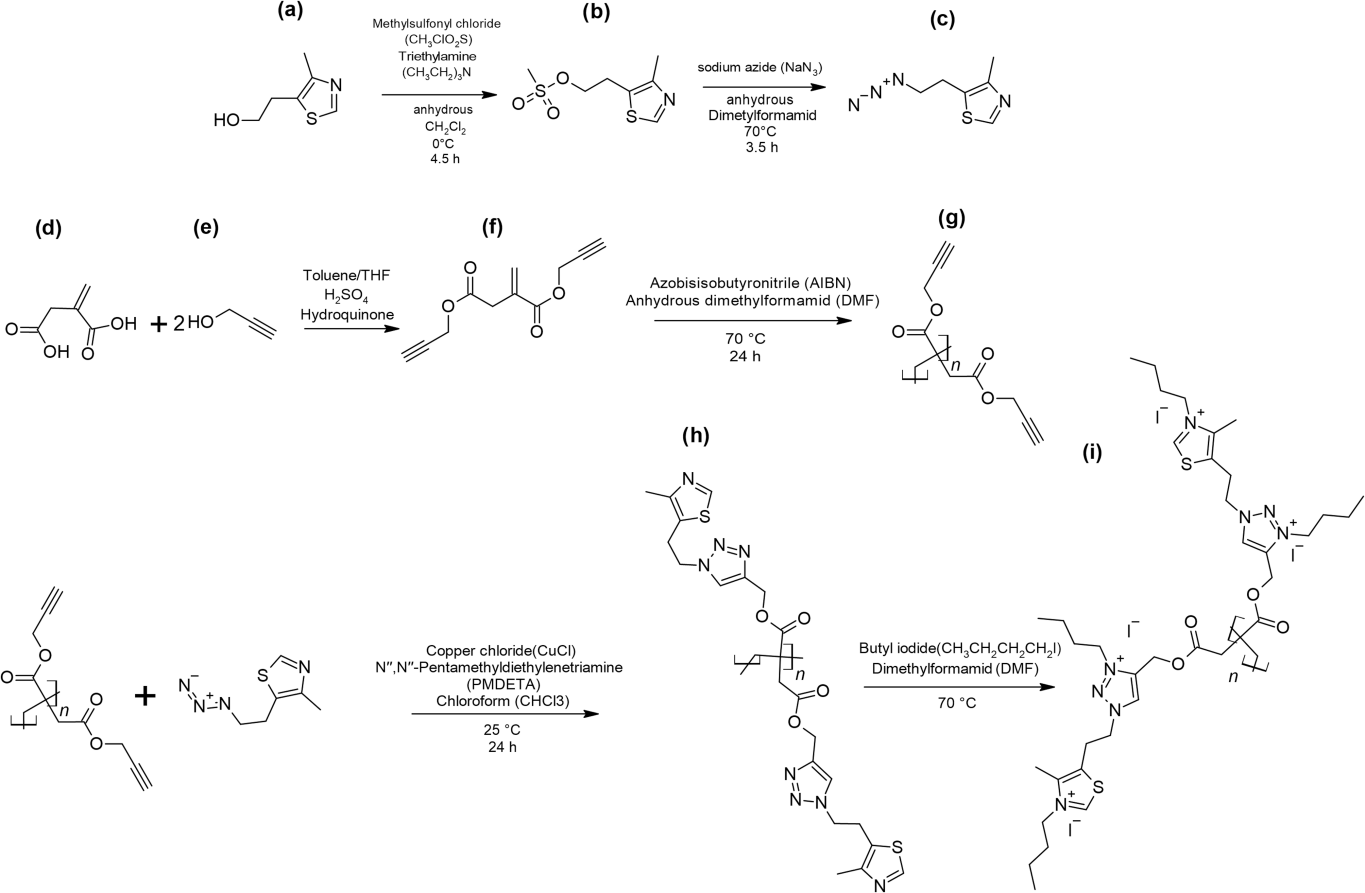
Synthesis of PTTIBuI, (a) 2-(4-methyl-1,3-thiazol-5-yl)­ethan-1-ol (5-(2-hydroxyethyl)-4-methylthiazole), (b) 2-(4-methyl-1,3-thiazol-5-yl)­ethylmethanesulfonate (M1), (c) 5-(2-azidoethyl)-4-methyl-1,3-thiazole (2-(4-methyl thiazole-5-yl) ethanol azide, (M2), d) 2-methylidenebutanedioic acid (itaconic acid), (e) 2-propyn-1-ol (propargyl alcohol), (f) di­(prop-2-yn-1-yl) 2-methylidenebutanedioate (di­(prop-2-yn-1-yl) itaconate, PrI), (g) poly­(di­(prop-2-yn-1-yl) itaconate) (PPrI), (h) poly­(bis­((1-(2-(4-methylthiazol-5-yl)­ethyl)-1H-1,2,3-triazol-4-yl)­methyl) itaconate) (PTTI), (i) poly­(5,5′-(((((2-methylenesuccinyl) bis­(oxy)) bis­(methylene)) bis­(3-butyl-1*H*-1,2,3-triazol-3-ium-4,1-diyl)) bis­(ethane-2,1-diyl)) bis­(3-butyl-4-methylthiazol-3-ium)) iodide (PTTIBuI).^11,32,34^

### Preparation of PLA + PTTIBuI Fibers

The solution for preparing fibers was made by dissolving 1 g of PLA in 6 mL of CH_2_Cl_2_. After the mixture was dissolved, 4 mL of DMF was added to the solution. This mixture was used to create the pure PLA fibers. For preparing PLA + PTTIBuI fibers, the solution was prepared as follows: 0.9 g of PLA was dissolved in 6 mL of CH_2_Cl_2_ and 0.1 g of PTTIBuI was dissolved in 4 mL of DMF. Both solutions were then mixed and homogenized using ultrasound for 15 min. The ratio between polymers was evaluated on previous experiments.^31^

### Electrospinning Process

The PLA + PTTIBuI fibers were prepared by an electrospinning process. The conditions were as follows: collector speed 1000 rpm, needle-to-collector distance 10 cm, voltage 16 kV, solution feed rate 1 mL/h, collection time 3 h, enclosure temperature 24 ± 1 °C and relative humidity 50 ± 5%. The resulting fibers were smooth and homogeneous, with diameters between 0.2–1 μm.

### Supercritical Process

PLA and PLA + PTTIBuI fibers were processed using a supercritical fluid process in a Speed SFE-4 device (Applied Separations Inc., USA). The treatment took place in an extraction cartridge at 200 bar and 60 °C for 3 and 6 h, depressurization rates were set at 80 bar/s. Samples were prepared repeatedly to verify the process, as some cartridges sealed poorly.

### Bacterial Strains

Potentially pathogenic strains *Acinetobacter baumannii* CCM7117 and methicillin-resistant *Staphylococcus aureus* (MRSA) CCM7111 were received from the Czech Collection of Microorganisms (Brno, Czech Republic). The strains were cultured at 37 °C on the plates with tryptone soya agar (HiMedia, India).^11^

### Methods of Characterization

For the characterization of the material, several methods were used. Synthesized PTTI and PTTIBuI were evaluated by nuclear magnetic resonance (1H NMR) and gel permeation chromatography (GPC). To observe surface changes, scanning electron microscopy with an atomic force module was used (SEM-AFM). Potential changes in chemical composition were evaluated by Fourier transform infrared spectroscopy (FTIR), and changes in crystallinity and phase composition were studied by X-ray diffraction (XRD) and differential scanning calorimetry (DSC).

^1^H NMR spectra of PTTIBuI were obtained in DMSO-*d*_6_ using a Bruker AVANCE III HD-400AVIII spectrometer operating at 400 MHz. The distribution of molecular weights of the polymer was measured by GPC using a chromatographic system Waters Division Millipore equipped with a refractive-index detector (Waters model 410). Dimethylformamide (99.9%, Aldrich) containing 0.1% of lithium bromide (≥99%, Aldrich), was used as the eluent at a flow rate of 1 mL min^–1^ at 50 °C. Styragel packed columns (HR2, HR3, and HR4, Waters Division Millipore) and poly­(methyl methacrylate) standards (Polymer Laboratories, Ltd.) between 2.4·10^6^ and 9.7·10^2^ g mol^–1^ were used.^11^

XRD analysis was conducted using a RIGAKU diffractometer using a Cu source (energy: 8.04 keV, wavelength: 0.15406 nm), operating at 40 kV and 40 mA, equipped with a Kβ filter and a scintillation detector. FTIR spectroscopy was performed using a Nicolet iS50 spectrometer on an ATR diamond crystal, with 62 scans recorded in the 400–4000 cm^–1^ (the mid-infrared range). The data were processed with ATR correction and baseline adjustment in OMNIC software. DSC was performed with a Setaram DSC 131 EVO over a temperature range of 0–200 °C at a heating rate of 5 °C/min. SEM was conducted using a JEOL JSM-7610F Plus (JEOL, Japan) with a field emission source, observing samples at 3 and 20 keV using secondary electron detection. Sputtering was performed with a Quorum Q150 V ES Plus system, applying a 2 nm gold coating at 30 mA. For surface topography evaluation, atomic force microscopy (AFM) was employed using a LiteScope in contact mode, with a scanning area of 20 × 20 μm^2^. The contact angle was measured using a Data Physics OCA 15 EC/B instrument using demineralized water (Milli-Q, 18.2 MΩ·cm; droplet volume 3 μL), recorded with a BU205 M camera with the high-performance UPHSV 205 upgrade.

### Determination of Inhibition of Biofilm Formation on Fibers

The effect of PLA, PLA + PTTIBuI, PLA + PTTIBuI (SFT—3h), and PLA + PTTIBuI (SFT—6h) fibers on biofilm formation was evaluated using the Crystal Violet Biofilm Assay.^11,35,36^ Fiber mats were cut into 1 × 1 cm^2^. Bacterial strains were cultured overnight at 37 °C with shaking at 120 rpm in tryptone soya broth (TSB). After incubation, bacterial suspensions were adjusted to 1 × 10^5^ CFU/mL in TSB. Fiber squares were placed in 48-well plates (1 square/well) with 1 mL of inoculum per well and incubated statically at 37 °C for 24 h. The supernatant was then aspirated, and the wells were washed twice with deionized water and dried at 60 °C for 15 min. Each well was stained with 1 mL of 0.1% crystal violet for 20 min at room temperature, then washed three times with deionized water and dried again at 60 °C for 15 min. Crystal violet was extracted with 96% ethanol (20 min, 200 rpm, room temperature), and 150 μL of the ethanol suspension was transferred to a 96-well plate for absorbance measurement at 584 nm using a microplate spectrophotometer. Biofilm reduction was calculated based on absorbance values, with each sample tested in six replicates.^37^ Statistical analysis was performed using one-way ANOVA with Tukey’s multiple comparison test, *p* ≤ 0.05 was considered significant. To further evaluate biofilm viability under identical cultivation conditions, the culture medium was removed. Then, 1 mL of 3-(4,5-dimethylthiazol-2-yl)-2,5-diphenyltetrazolium bromide (MTT) reagent (0.5 mg mL^–1^ in phosphate-buffered saline) was added to each well, and the samples were incubated at 37 °C for 2 h. Following aspiration, the formed formazan was dissolved in 1 mL of dimethyl sulfoxide (200 rpm, 20 min), and the absorbance was recorded at 550 nm. Cell viability was calculated as a percentage relative to the PLA control, and the data are presented as mean values.

## Results and Discussion

First, PTTIBuI was prepared and characterized using ^1^H NMR and GPC, which confirmed its structure. The results are shown in the Supporting Information (Figure S1). The PTTIBuI/PLA mixture was then electrospun into fibers. Films of PLA and PLA+PTTIBuI fibers with a uniform thickness of 8–10 μm were prepared on reinforced aluminum foil by electrospinning. These fibers were then subjected to supercritical and thermal processing, after which the changes were evaluated.

### Scanning Electron Microscopy and Elements Mapping

Figure 2a–c display morphology of PLA fibers before and after supercritical treatment. Figure 2a shows prepared PLA fibers with diameters ranging from 400 to 700 nm. Figure 2b depicts PLA fibers after thermal treatment in the SFT oven without supercritical CO_2_, it can be seen that polymeric fibers partially melted together, resulting in higher diameter of fibers. Figure 2c depicts PLA fibers after supercritical treatment. The melting of the fibers is lower due to the dissolution of PLA in scCO_2_. The PLA fibers after supercritical treatment shows increase in surface area by “wrinkling” caused by melting, crystallization and dissolution of polymer.

**Figure 2:**
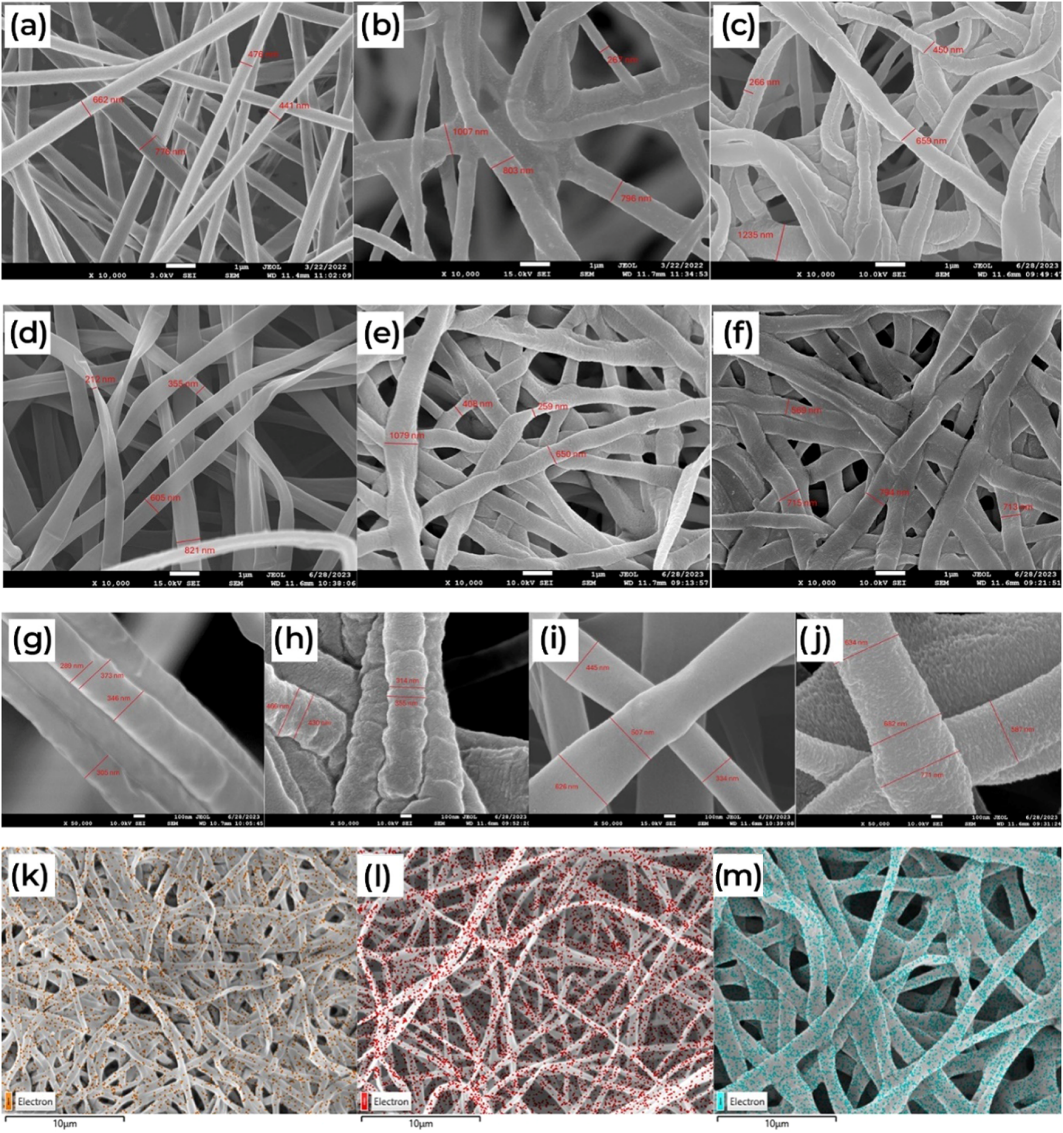
(a) pure PLA fibers before supercritical treatment, (b) pure PLA fibers treated only with temperature 60 °C for 3 h, (c) pure PLA fibers after supercritical treatment 3h, 20 MPa, 60 °C, (d) PLA+PTTIBuI fibers before treatment, (e) PLA + PTTIBuI fibers after supercritical treatment 3h, 20 MPa, 60 °C, (f) PLA + PTTIBuI fibers after supercritical treatment 6h, 20 MPa, 60 °C, (g) detail of pure PLA fibers before supercritical treatment, (h) detail of pure PLA fibers after supercritical treatment 3h, 20 MPa, 60 °C, (i) detail of PLA + PTTIBuI fibers before treatment, (j) detail of PLA + PTTIBuI fibers after supercritical treatment 3 h, 20 MPa, 60 °C, (k) iodine on PLA + PTTIBuI fibers after supercritical treatment for 6 h, (l) iodine on PLA + PTTIBuI fibers, (m) iodine on PLA + PTTIBuI fibers after supercritical treatment for 3h.

Figure 2d–f show PLA + PTTIBuI fibers before and after supercritical treatment for 3 and 6 h. There are no significant differences in diameter between pure PLA fibers and PLA + PTTIBuI fibers, both measuring in similar diameters ∼400–800 nm. The only difference between PLA fibers and PLA + PTTIBuI fibers is in shape of the fibers, the PLA + PTTIBuI fibers have more flattened shape. Like PLA fibers, PLA + PTTIBuI fibers exhibit similar surface changes after supercritical treatment.

In addition, the appearance of the fibers before SFT treatment and their diameters (Figure 2a,d) were in good agreement with, for example, fibers from polylactide-*co*-ethylene carbonate copolymer, which have similar diameters. Both PLA and PLA + PTTIBuI fibers are almost free of defects, although the concentration of PLA and PLA + PTTIBuI used for fiber preparation was approximately 10% (w/v), while in the case of polylactide-*co*-trimethylene carbonate copolymer, the optimal concentration was 12% (w/v).^38^

Figure 2g–j provides detailed images of PLA and PLA + PTTIBuI fibers before and after supercritical treatment. The comparison shows changes in the surface morphology due to the crystallization and polymer dissolution in scCO_2_. The increased surface area may enhance the fibers’ antimicrobial effect.^22,39,40^

Figure 2k–m shows the distribution of iodine in PLA + PTTIBuI fibers. Iodine was selected as the indicator for the presence of PTTIBuI because the response intensity of other elements such as nitrogen was barely detectable. The figures indicate that there are no significant changes or disappearance of iodine in the PTTIBuI fibers after supercritical treatment.

### Fourier-Transform Infrared Spectroscopy

Fourier-transform infrared spectroscopy (FTIR) with attenuated total reflectance (ATR) was used to record the spectra of pure PLA fibers (Figure 3a), PLA + PTTIBuI fibers, and pure PTTIBuI powder; all wavenumber bands and their corresponding chemical bonds are shown in Table 1. The characteristic bands for PTTIBuI are =C–H and −C–N+, however they are not clearly visible in those polymer mixtures probably due to the low content of PTTBuI comparing to PLA. No occurrence of a new bond is evident from Figure 3a. It means that the chemical entity of both polymers did not change, and they are present as blends.

**Figure 3:**
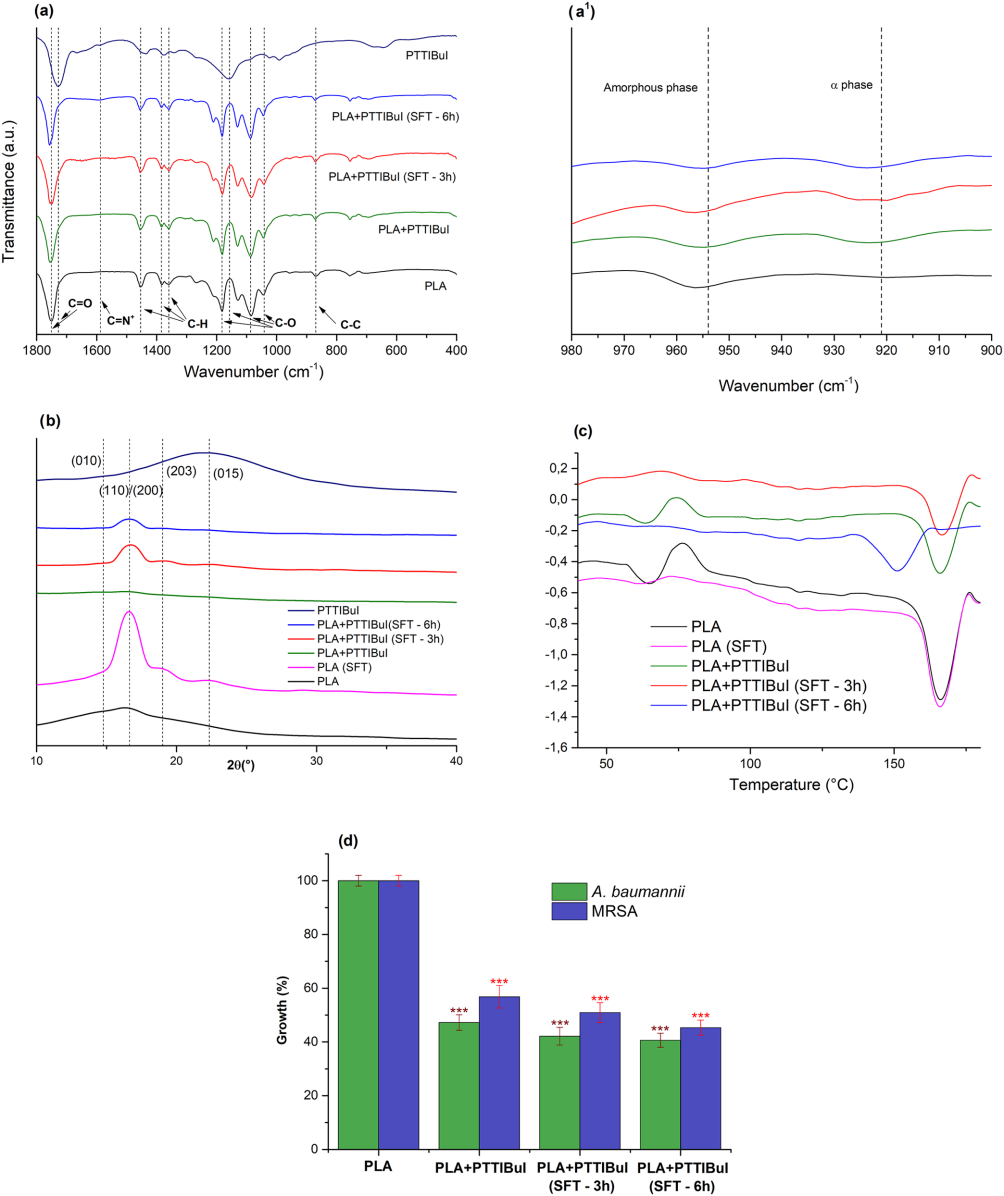
(a) FTIR spectra of PLA and PLA + PTTIBuI fibers, (a1) detail of FTIR spectra of PLA and PLA + PTTIBuI fibers with focus on the crystallinity phase, (b) XRD patterns of PLA and PLA + PTTIBuI fibers, (c) DSC patterns for PLA and PLA+PTTIBuI fibers, (d) biofilm inhibitory and SFT effect of PLA + PTTIBuI, significant differences compared to the pure PLA control group are indicated by asterisks: *** *p* < 0.001, as determined by Tukey’s test.

**Table 1:** FTIR Spectra of PLA and PLA + PTTIBuI—Wavenumberswa and Corresponding Bonds

PTTIBuI	wavenumber cm^–1^	PLA	wavenumber cm^–1^
≡C–H	3282	–C–H	2994
–C–H	2960	–C–H	2946
–C–H	2935	C=O	1751
–C–H	2867	–CH_3_	1450
C=O	1727	C–H	1382
–C–N^+^	1587	C–H	1355
–CH_3_	1432	C–O–C	1263
C–O–C	1268	C–O–C	1180
C–O–C	1155	C–O–C	1128
C–C	991	C–O–C	1081
≡C–H	687	C–O–C	1039
≡C–H	638	the skeletal stretching and CH_3_ rocking	954, 921
		C–C	871
		C–C	754

### X-ray Diffraction

XRD patterns of pure PLA fibers as prepared and after SFT treatment, PLA + PTTIBuI fibers, and pure PTTIBuI powder are shown in Figure 3b. It can be seen that pure PLA fibers and PTTIBuI powder are characterized by broad halos, which indicates that both polymers are in an amorphous state.^2,11,41^ The amorphous state of electrospun PLA fibers was described in many studies.^42–45^ This is consistent with the rapid solidification of the polymer jet during electrospinning that limits chain ordering. In the case of PLA + PTTIBuI fibers, a dominant amorphous state was also clearly determined.

After supercritical processing of pure PLA fibers, characteristic high-intensity diffraction peaks at 16.9° and 19.1° and a low-intensity peak at 22.5°, typical of PLA, appeared in the XRD pattern. They correspond to the crystal planes (200/110), (203) and (015) of the α-form of the crystalline phase. This suggests that during SFT, crystalline regions are formed in the initially amorphous PLA to form semicrystalline PLA (Figure 3b). It is also confirmed by the FTIR spectra (Figure 3a, with a detail in Figure 3a1), which showed the presence of wavenumber bands of 954 and 921 cm^–1^, corresponding to the presence of amorphous PLA and α or α‘-forms of crystalline PLA.^45–48^ This was observed for all samples treated with SFT. It appears that the plasticizing effect of scCO_2_ was exerted, increasing chain mobility and allowing PLA chains to align during processing. Based on the shape and intensity of the characteristic X-ray diffraction peaks, it is clear, that in the case of PLA + PTTIBuI, the crystallization ability of PLA decreased over time during the SFT process, however, the formation of crystalline domains persists.^11,47^

### Differential Scanning Calorimetry

The thermal behavior of PLA, PLA (SFT—3h), PLA + PTTIBuI, PLA + PTTIBuI (SFT—3h) and PLA + PTTIBuI (SFT—6h) was further studied by DSC (Figure 3c). The glass transition temperature (*T*_g_), the cold crystallization temperature (*T*_cc_), the melting temperature (*T*_m_) and the melting enthalpy (Δ*H*_m_) of each material are shown in Table 2. The degree of crystallinity (*X*_c_) was calculated by subtracting the enthalpy of crystallization (Δ*H*_cc_) from the melting enthalpy (Δ*H*_m_) and dividing it by the melting enthalpy corresponding to a single PLA crystal (Δ*H*_f_ = 93.6 J g^–1^).^2,49^ Both PLA and PLA + PTTIBuI fibers before SFT treatment showed typical DSC curves, that included *T*_g_ around 65 and 64 °C, respectively, indicating also probable physical aging. They were followed by a cold crystallization region with *T*_cc_ values of 77 and 75 °C and then a melting transition at approximately 166 °C. The Δ*H*_cc_ value for an amorphous polymer is practically the same as the value of Δ*H*_m_ and the difference between these enthalpies gives the initial crystallinity of the fibers.^11^ In the case of an amorphous polymer, it is equal to 0%, which was estimated in the case of PLA and PLA + PTTIBuI. However, after 3 h of SFT treatment, PLA crystallizes and does not show cold crystallization transition, but crystalline melt with *T*_m_ of 166.0 °C. The enthalpy is 41.3 J/g, which corresponds to approximately 44% crystallinity. In the case of PLA + PTTIBuI after 3 h of SFT, the cold crystallization region disappears and only *T*_g_ and *T*_m_ are visible on the curve at around 62.6 and 167.0 °C, respectively. The disappearance of the cold crystallization peak suggests that the treated fibers are already largely crystalline before the DSC heating scan, i.e., scCO_2_ induces crystallization during the treatment (in agreement with the XRD results), so that no further cold crystallization occurs upon heating. After 6 h of SFT, however, a relatively significant decrease in crystallinity (*X*_c_ = 26%) can be observed, which is accompanied by a significant decrease in *T*_m_ to 151 °C. This indicates that SFT process at longer times provokes the less stable α’-form of PLA. The decrease in melting temperature is attributed to the plasticizing action of scCO_2_ on PLA: dissolved CO_2_ increases chain mobility and relieves internal stresses, promoting the formation of thinner and less perfect lamellar crystals that melt at a lower temperature.

**Table 2:** Parameters of DSC, Contact Angle Measurements and Inhibition Results

parameter	PLA	PLA (SFT—3h)	PLA + PTTIBuI	PLA + PTTIBuI (SFT – 3h)	PLA + PTTIBuI (SFT – 6h)
*T*_g_ (°C)	65.0	62.1	63.5	62.6	60.4
*T*_cc_ (°C)	77.0	-	74.5	-	-
*T*_m_ (°C)	166.0	166.20	166.0	167.0	151.0
Δ*H*_m_ (J/g)	47	41	42	38	24
*Χ*_c_ (%)	0	44	0	41	26
Contact angle (°)	123 ± 10	61 ± 16	116 ± 3	109 ± 11	86 ± 11
Inhibition (%)					
*A. baumannii*	-	-	52.8 ± 2.9	57.9 ± 3.3	59.4 ± 2.6
MRSA	-	-	43.2 ± 4.2	49.1 ± 3.7	54.7 ± 2.8

These results are in agreement with FTIR measurements, where the presence of amorphous and crystalline (α or α’) phases are confirmed by the presence of bands at 954 and 921 cm^–1^ in the FTIR spectrum corresponding to the skeletal stretching C–C and CH_3_ rocking (Figure 3a,a1). And it is also supported by the XRD patterns, where a change in the intensity of the peaks corresponding to the α or α′ crystalline phases is visible. The presence of PTTIBuI thus significantly affects the morphology of PLA. It is also clear that while 3h SFT was beneficial for increasing the crystallinity of the sample, 6h was already accompanied by a decrease in these properties and led to lower quality samples. It is known that the α-form is formed during crystallization at higher temperatures,^47^ but here it is clear that supercritical conditions allowed the formation of this form under milder temperature conditions.

Figure S2 demonstrates a detailed view of PLA+PTTIBuI fibers before and after SFT treatment (3 and 6 h) using SEM. It is clear that porosity and roughness appear in the fiber.^11,50^ The treated fibers also withstood contact-mode AFM scanning without tip-induced damage, unlike the untreated fibers (Figure S3), suggesting improved mechanical robustness; quantitative nanomechanical testing was beyond the scope of this study.

### Surface Tension-Contact Angle Measurement

Table 2 shows the droplet analysis results for fiber samples tested using demineralized water. Examples of water droplet contact angle measurements on these materials are shown in Figure 4. The prepared fibers (PLA and PLA + PTTIBuI) are hydrophobic. After supercritical treatment, a decrease in hydrophobicity is observed for PLA and PLA + PTTIBuI samples. This may be caused by the higher surface and the ability of C=O groups to interact with water.

**Figure 4:**
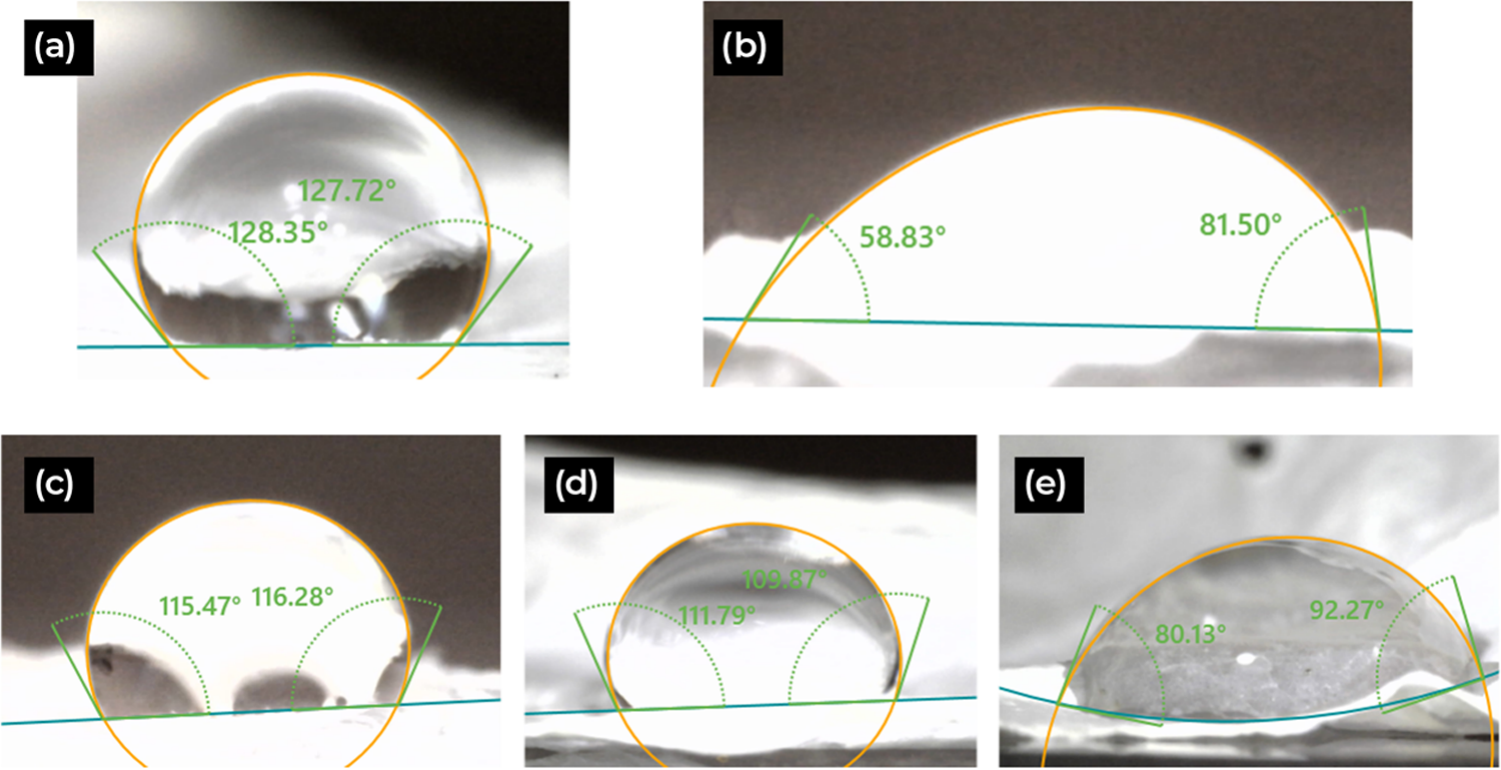
Example of the contact angle of water drop on: (a) Surface of PLA fibers, (b) surface of PLA fibers after supercritical treatment, (c) surface of PLA + PTTIBuI fibers, (d) surface of PLA + PTTIBuI fibers after supercritical treatment for 3 h, (e) surface of PLA + PTTIBuI fibers after supercritical treatment for 6 h.

The wettability results can be interpreted within the Wenzel and Cassie–Baxter frameworks. The untreated electrospun mats are highly porous, so the high apparent contact angle of pristine PLA (123°)—well above the intrinsic value reported for flat PLA (∼80°)^11,51^ is consistent with a Cassie–Baxter state, in which air trapped between the fibers forms a composite solid–air interface that supports the droplet. After scCO_2_ treatment, SEM reveals surface wrinkling, partial melting and fiber fusion, which collapse this trapped-air structure and allow water to penetrate the texture, driving a transition toward a Wenzel wetting regime. Because the intrinsic contact angle of PLA is below 90°, wetting in the Wenzel regime lowers the apparent angle, consistent with the observed decrease to 61°. The same trend is seen for PLA + PTTIBuI (116° → 86°), where the cationic thiazolium/triazolium groups further reduce the intrinsic surface energy. The pronounced drop in contact angle after treatment therefore reflects both a topographical contribution (loss of trapped air, Cassie–Baxter → Wenzel) and a chemical contribution (increased accessibility of polar groups). A quantitative determination of the roughness factor (*r*) and the solid fraction (*f*_s_) was beyond the scope of this study; the models are used here to interpret the wetting behavior qualitatively.

### Antimicrobial Tests

The inhibitory effect of all samples with PLA + PTTIBuI is compared to pure PLA (100% biofilm formation) (Table 2), with a significant reduction of biofilm (40–60%). Both bacterial strains showed significant inhibitory effects on biofilm formation (*p* < 0.05). A more pronounced effect was observed in the *A. baumannii* strain for all samples (with a reduction effect of ca. 50–60%), while the effect on MRSA was slightly lower. Thus, it can be said that antimicrobial polymer is affecting more Gram-negative bacteria than Gram-positive. Samples with SFT showed a higher inhibitory effect compared to those without SFT, and the influence of SFT increased over time, being more pronounced at 6 h than at 3 h. Increased antimicrobial activity is a direct consequence of a higher surface area with more antimicrobial groups (thiazolium and triazolium) exposed to interact with bacteria.^32^ In a previous study, the PTTIBuI was tested against *S. aureus*, *Staphylococcus epidermidis*, *Escherichia coli* and *Pseudomonas aeruginosa* bacteria. It was very effective against Gram-positive bacteria with minimum inhibition concentration (MIC) values of 8 and 10 μg/mL but almost ineffective against Gram-negative (MIC > 5000 μg/mL). With the SFT process the active groups of poly­(itaconate derivative) are more accessible and effective. To further verify the antibiofilm efficiency, the inhibitory effect of SFT-treated PLA + PTTIBuI samples on biofilm cell viability was evaluated. For *Acinetobacter baumannii* strain, the viability inhibition reached 56% for the PLA + PTTIBuI (SFT—3h) sample and increased to 65% for the PLA + PTTIBuI (SFT—6h) sample. In the case of MRSA strain, this value increased markedly from 47% (SFT—3h) to 62% (SFT—6h). These results are in good agreement with the biofilm biomass reduction for both strains. The increased antimicrobial activity could be related to a higher surface area of the material and the subsequent availability of antimicrobial groups (thiazolium and triazolium) for interaction with bacteria.^52^

The antibiofilm performance of the fibers prepared in this work is comparable to other PLA-based antimicrobial systems, although it should be noted that the reported efficacy is highly dependent on the assay used. Many of the most effective systems use leachable or metal-based agents: PLA films grafted with silver nanoparticles by γ-irradiation achieve more than 99% reduction of *E. coli* and *S. aureus* while reducing the contact angle from 94.5° to 64.1°.^25^ PLA composites containing fillers modified with silver or quaternary ammonium ions develop their activity gradually due to the slow release of the biocide during polymer degradation.^2^ The contact-active systems derived from itaconic acid achieve high reductions only upon the addition of a releasing functional group; for example, electrospun PLA fibers bearing both cationic triazolium and N-halamine groups achieve up to a 5-log (≈99.999%) reduction, but the strongest effect depends on the release of oxidative chlorine from the N-halamine.^31^ In our previous study, a PLA surface treated with SFT modified with cationic polymethacrylate (PMTABuI) inhibited biofilm formation by 77–90% and reduced biofilm viability by 82–92% against the same strains, although the methacrylate modifier was found to be washed out in aqueous media.^11^ In comparison, the PLA + PTTIBuI fibers described here provide a moderate but sustained, nonleachable effect (≈50–60% biomass and up to 62–65% MTT viability reduction) against *A. baumannii* and MRSA, based solely on the contact-active cationic thiazolium and triazolium groups of the biobased biodegradable polyitaconate, without any metal, releasable biocide, or ionization treatment. Importantly, the supercritical CO_2_ step enhances this contact-active effect through a purely physical surface restructuring—for example, biofilm inhibition increased from 52.8% to 59.4% against *A. baumannii* and from 43.2% to 54.7% against MRSA after 6 h of SFT—using a single, nontoxic, fully recoverable medium and avoiding the organic solvents, corrosive reagents, and high-energy radiation required by alternative routes. The SFT step is readily tunable through pressure, temperature, and treatment time, offering a sustainable and scalable means of adjusting surface area, crystallinity, and hydrophilicity, and thereby the accessibility of the antimicrobial groups.

## Conclusion

This study investigated the effects of supercritical fluid treatment (SFT) on PLA and PLA + PTTIBuI fibers, prepared via electrospinning and treated with supercritical CO_2_. Supercritical CO_2_ treatment reduced the water contact angle of PLA from 123° to 61° and of PLA + PTTIBuI from 116° to 86° after 6 h, confirming a shift from hydrophobic to hydrophilic behavior. Biofilm inhibition by PLA + PTTIBuI increased with treatment time, reaching 50–60% against *A. baumannii* and 43–55% against MRSA, with the strongest effect after 6 h of SFT; the MTT viability assay confirmed this trend (up to 65% and 62% viability reduction, respectively).

X-ray diffraction (XRD) revealed the amorphous state of the PTTIBuI polymer, PLA and PLA + PTTIBuI fibers, but after SFT treatment the crystalline phase developed. Surface morphology showed changes and increased porosity and roughness due to SFT. Drop analysis demonstrated a shift from a hydrophobic to hydrophilic nature after 6 h of treatment. Antimicrobial testing showed significant biofilm inhibition in PLA + PTTIBuI fibers, slightly more against *A. baumannii* (50–60%), with enhanced effects observed after 6 h of SFT. The supercritical treatment improves the antimicrobial effect but also reduces the difference in inhibitory effect between Gram-negative and Gram-positive bacteria. This means that the use of the SFT method could increase the effectiveness of materials for the inhibition of bacterial infections. Overall, supercritical fluid treatment improved antimicrobial properties and altered structural and thermal characteristics, highlighting the potential of these modified fibers in biomedical applications, particularly for wound care and infection prevention.

In addition, SFT appears to be a suitable technology that allows the adjustment of process conditions (temperature, pressure, amount, and time) to achieve the optimal degree of crystallinity, porosity/roughness, surface area, and hydrophilicity. Importantly, this antibacterial enhancement is achieved without metal nanoparticles or leachable biocides, using a biodegradable itaconic acid-derived polymer and a green, residue-free scCO_2_ process. This positions the approach as a sustainable route to nonleaching, contact-active antimicrobial PLA fibers for applications such as wound dressings, medical bandages, and air-filtration materials.

The authors would like to thank P. Peikertová for the FTIR measurements, R. Gábor for the SEM and AFM measurements, S. Holešová for DSC measurements, and M. Hundáková for the XRD measurements.

## Supplementary Material



## Notes

^1^H NMR spectra of PTTABuI in DMSO-*d*_6_ with caption, GPC analysis results (Figure S1); (a) PLA+PTTIBuI, (b) PLA+PTTIBuI after 3 h of supercritical processing, (c) PLA+PTTIBuI after 6 h of supercritical processing showing scanning electron microscopy images (Figure S2); (a) PLA+PTTIBuI after 3 h of supercritical processing, (b) PLA+PTTIBuI after 6 h of supercritical processing with caption showing atomic force microscopy (AFM) measurement results (Figure S3) (PDF)
