# Supplementary material for: Tailoring the Surface of Polylactide/Poly(itaconate derivative) Fibers: A Path to Antibacterial Enhancement
Source: ACS Omega. 2026 Jul 28;11(31):46723–33. doi: 10.1021/acsomega.6c04582 (PMC13470690; doi:10.1021/acsomega.6c04582)
Supplement: Supplementary file 1 [file ao6c04582_si_001.pdf]

Tailoring the Surface of Polylactide/Poly(itaconate derivative) Fibers: A Path to Antibacterial Enhancement

Jakub Zágora[1,2*], Zuzana Rybková[1,3], Kateřina Škrlová[4], Kateřina Malachová[1,3], Alexandra Muňoz-Bonilla[5], Marta Fernández-Garcia[5], Daniela Plachá[6,7*]

[1]Nanotechnology Centre, CEET, VSB-Technical University of Ostrava, 17. listopadu 2172/15, 708 00 Ostrava- Poruba, Czech Republic

[2]Faculty of Materials Science and Technology, VSB-Technical University of Ostrava, 17. listopadu 2172/15, 708 00 Ostrava- Poruba, Czech Republic

[3]Department of Biology and Ecology, Faculty of Science, University of Ostrava, Chittussiho 10, 71000 Ostrava, Czech Republic

[4]Departement of Chemical Biology, Faculty of Science, Palacký University Olomouc, 17. listopadu 1192/12, 779 00 Olomouc, Czech Republi

[5]Institute of Science and Technology of Polymers, CSIC, Juan de la Cierva 3, 28000 Madrid, Spain

[6]CATRIN-RCPTM, Palacký University Olomouc, 17. listopadu 241/27, 779 00 Olomouc, Czech Republic

[7]Department of Epidemiology and Public Health, Faculty of Medicine, University of Ostrava, Syllabova 19, 703 00, Ostrava – Vítkovice, Czech Republic

Corresponding authors*: daniela.placha@upol.cz, jakub.zagora@vsb.cz

NMR

^{1}H NMR (300 MHz, DMSO-d6): δ = 10.3 (br, 1H; N-C(H)-N, imidazolium), 9.25 (br, 1H; N-C(H)-S, thiazolium), 5.5 (br, 1H; -CH-O-), 5.0 (br, 2H; C-CH_2-N), 4.75 (br, 2H; N^+-CH_2-C, imidazolium), 4.50 (br, 2H; N^+-CH_2-C, thiazolium), 3.75 (br, 2H; C-CH_2-C), 3.50 (br, 2H; COO-CH_2-C), 2.50 (br, 3H; CH_3, s, 1 H, residual DMSO-d_6), 1.75 (br, 2H; C-CH_2-C), 1.30 (br, 2H; C-CH_2-C), 0.80 (br, 3H; CH_3)

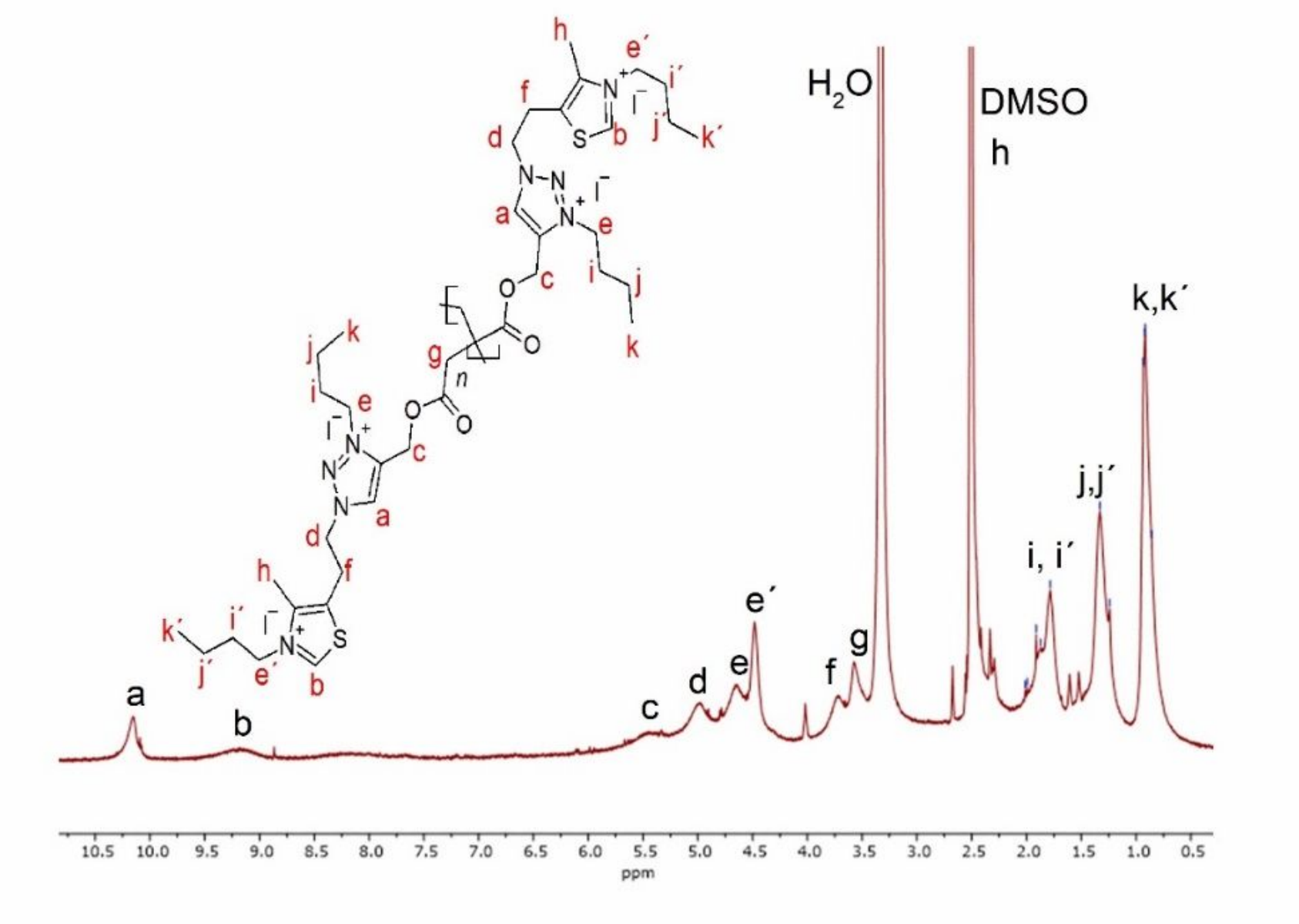

Figure S1. ^{1}H-NMR spectra of PTTABuI in DMSO-d6.

GPC

The average molecular weight (M_n) and polydispersity index (PDI) of the synthetized polymer before and after quaternization were determined by GPC. Due to the insolubility of the PTTIBuI polymer in dimethylformamide, only the unmodified PPrI polymer was measured by GPC. The results for this are Mn= 6,700 Da PDI: 1.6.

Electron microscopy

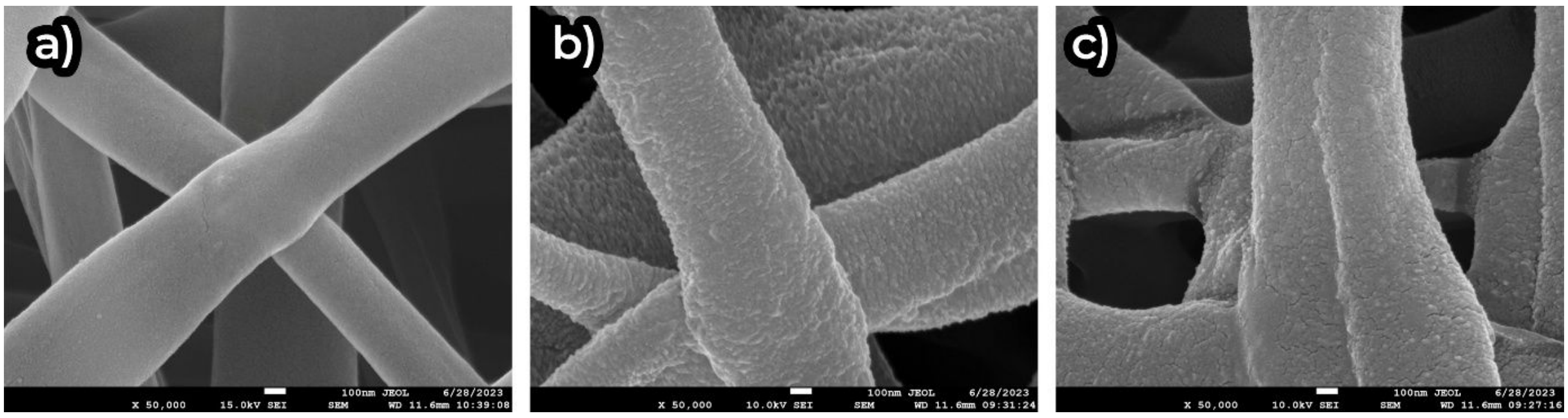

Figure S2. a) PLA+PTTIBuI, b) PLA+PTTIBuI after 3h of supercritical treatment, c) PLA+PTTIBuI after 6h of supercritical treatment

Atomic force microscopy

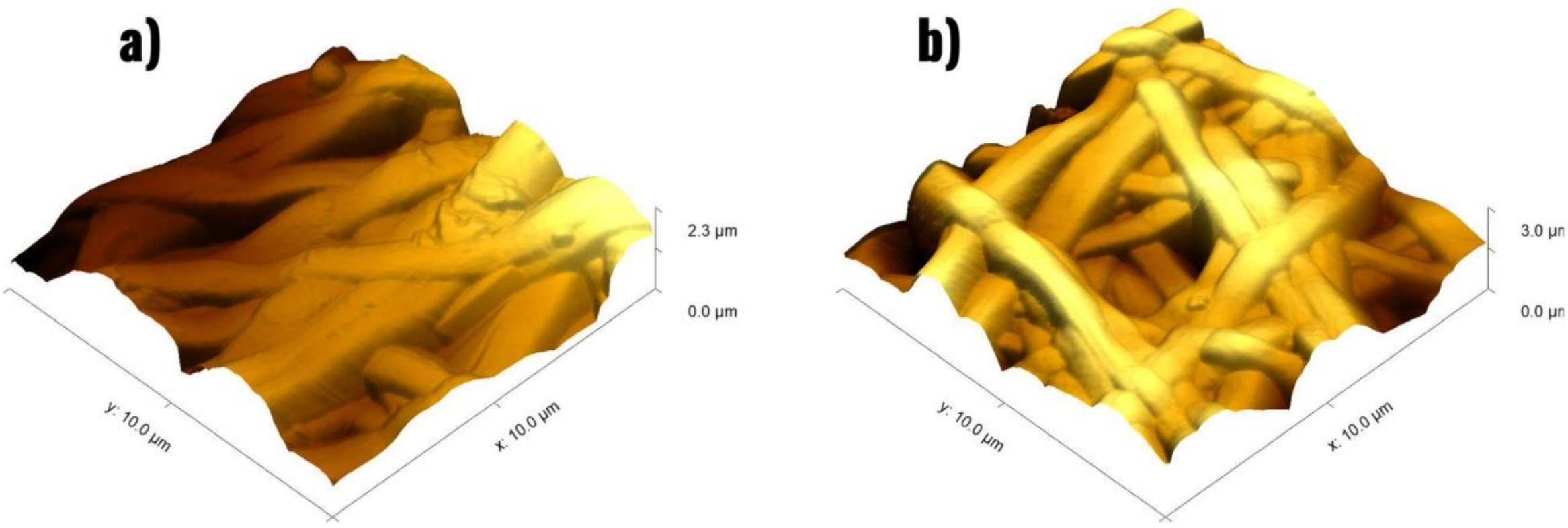

Figure S3. a) PLA+PTTIBuI after 3h of supercritical treatment, b) PLA+PTTIBuI after 6h of supercritical treatment

Figure S3 shows the surface morphology of PLA+PTTIBuI fibers after supercritical treatment. The fibers before supercritical treatment could not be measured due to their charge and high fragility. It is a confirmation of the better mechanical quality of
